# Could sleep engineering be used to combat PTSD and depression?

**DOI:** 10.1371/journal.pbio.3003633

**Published:** 2026-02-23

**Authors:** Penelope A. Lewis, Mahmoud E. A. Abdellahi

**Affiliations:** 1 Psychology Department, Cardiff University Brain Research Imaging Centre (CUBRIC), Cardiff University, Cardiff, United Kingdom; 2 Faculty of Computers and Artificial Intelligence, Cairo University, Giza, Egypt

## Abstract

Could sleep engineering be developed to provide a drug-free, non-invasive avenue to treat depression and post-traumatic stress disorder? This Perspective proposes using machine learning with EEG signals to develop and optimize this type of intervention.

## Teaser

Sleep engineering could be developed to provide a drug-free, non-invasive avenue to treat depression and post-traumatic stress disorder. Such an intervention would be greatly aided by the sophisticated detection of memory reactivations using machine learning classifiers.

Sleep engineering, in which memories and brain oscillations are intentionally manipulated during sleep in order to influence health and cognition, provides a promising new therapeutic toolkit. In a form of sleep engineering called targeted memory reactivation (TMR), sounds are paired with learned information during wake, then softly replayed during subsequent sleep to cue the brain to reactivate neural representations of the learned information. This method has already provided three potential avenues for psychiatric treatment by strengthening memories, altering their emotionality, and helping them to integrate into existing cognitive schemas [1]. We propose that using electroencephalogram (EEG) classifiers to study emotional memory reactivation, and the ways in which this is altered by TMR in patients, could enable better understanding and control of such interventions for conditions such as depression and post-traumatic stress disorder (PTSD). Careful thinking around dosage, adverse effects, and safeguarding will be essential.

**Fig 1:**
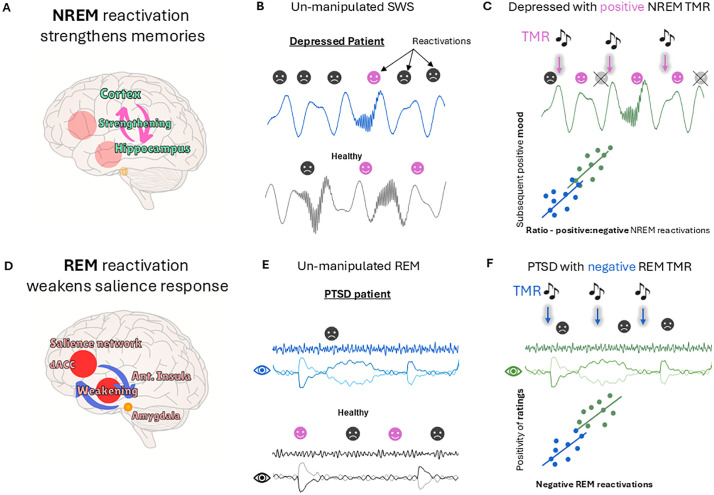
Hypothetical method for normalizing aberrant emotional memory processing in sleep. **A**. Non-rapid eye movement (NREM) sleep reactivation strengthens cortico–hippocampal interplay. **B**. We speculate that individuals with depression have more negative reactivation during NREM sleep, which strengthens negative memories. **C**. Targeted memory reactivation (TMR) of positive memories in NREM sleep primes these memories such that they outcompete negative memories, giving a higher ratio of positive to negative reactivation. This positively correlates with better subsequent mood. **D**. Rapid eye movement (REM) sleep reactivation weakens the salience network–amygdala interplay. **E**. We speculate that individuals with post-traumatic stress disorder (PTSD) have less healthy negative reactivations in REM sleep. **F**. Negative TMR in REM sleep increases healthy negative REM reactivation, reducing the negativity of targeted memories. Note that this figure presents a series of hypotheses and all data are hypothetical.

The most direct application of sleep engineering to psychiatric conditions to date involves strengthening the memory of a therapy using TMR during non-rapid eye movement (NREM) sleep (Fig 1). This first method already improved clinical outcomes via eye-movement desensitization and reprocessing therapy in individuals with PTSD [2], and via imagery rehearsal therapy in individuals with nightmares [3]. In a closely related second avenue for treatment, NREM TMR was used to update negative memories by increasing their positive associations [4,5], rendering the memories less negative, even when delivered entirely in sleep [1,5]. In a quite different third avenue for treatment, TMR in rapid eye movement (REM) sleep was used to directly attenuate the negativity associated with an upsetting memory [6] (Fig 1). Reactivation in REM sleep gradually decoupled negative content from core trauma memories as if they were being retrieved during a ‘safe’ therapy session. This decoupling is thought to occur because norepinephrine, a neurotransmitter that modulates bodily arousal responses, is almost absent during REM sleep, so arousal cannot be expressed physiologically. Although only the first of these three methods has been tested in patients, and TMR results are often inconsistent across studies, we nevertheless feel that all three methods provide promising potential avenues for the development of novel treatments for PTSD and depression.

Individuals with depression often show pathological negative rumination, which we speculate could be associated with excessive replay of negative memories during sleep (Fig 1). Consistent with this, such individuals typically feel worse in the morning, presumably having strengthened their negative memories and renewed their overall negative schema and outlook during the night. We propose that repeated NREM TMR of positive memories could be used to disrupt this maladaptive cycle and prevent the over-consolidation of negative memories. Because the resources recruited by TMR are limited [7] and would be tied up reactivating positive memories, this would provide a two-pronged approach for treating depression by strengthening the positive while simultaneously preventing over-strengthening of the negative.

Classifiers, which use machine learning to detect subtle signals in noisy EEG data, provide an invaluable tool for detection, quantification, and characterization of TMR-elicited memory reactivation during sleep. Such classifiers can detect spontaneous reactivation [8] in un-disturbed sleep and will allow us to determine how reactivation of negatively versus positively toned memories differs in individuals with PTSD or depression compared with healthy individuals. These helpful tools will let us search for relationships between the efficacy of TMR and target clinical outcomes; for instance, testing our hypothesis that boosting positive memory reactivation during NREM sleep can decrease negative memory reactivation and improve subsequent mood (Fig 1).

Until recently, the EEG classifiers used to detect reactivation in sleep ignored the emotional content of the memories. However, we have now developed a novel classifier pipeline that can be pre-trained to detect whether reactivations occurring after TMR cues are negative or neutral [9]. This will allow us to determine how processing of memories in sleep differs between healthy participants and patients. Understanding the characteristics of emotional reactivation, such as when it occurs most strongly or which neural signatures predict better therapeutic responses, could inform how we tailor TMR for different patients, potentially adjusting factors like cue timing, frequency, or intensity to optimize emotional processing during sleep.

While NREM TMR normally strengthens memories, it can also weaken them when specifically targeted at the down-state of slow oscillations [10,11]. Down-state TMR could therefore potentially provide an additional mechanism for gradually eroding core information in unwanted negative memories. This weakening may be due to down-state TMR causing reactivation at a phase of the oscillation that is not beneficial for consolidation. After reactivating, engrams might enter a refractory period and therefore would not reactivate during the subsequent optimal up-phase, so that the overall impact would be negative. Down-state stimulation also disrupts slow oscillations and sleep spindles, both of which are important for memory consolidation. Detecting down-state reactivations with EEG classifiers and testing for correlations with mood outcomes should help us to understand their true impact.

The potential for combining different forms of sleep engineering within an individual to treat different aspects of a psychiatric illness remains largely unexplored. An example would be delivery of both positive NREM TMR and negative REM TMR on the same or adjacent nights. This could provide a triple benefit by both strengthening positive memories and preventing over-consolidation of negative memories in NREM, while also directly reducing the negativity of negative memories in REM (Fig 1). However, there is a danger that stimulation in these two sleep stages could interfere with each other. Because the influence of TMR is thought to bleed between sleep stages [1], carefully planned studies will be needed to determine whether it is beneficial to deliver both NREM and REM TMR in the same night or even in the same week/month. It may also be possible to tailor interventions by targeting some memories more than others, depending on how strong they are already, and how much additional “help” they need.

Most sleep engineering research has been limited to a single night because the interventions are typically applied in the lab and require experimenters to be awake and actively delivering them. The recent advent of wearable devices that can perform TMR in the home is rapidly changing this picture [12]. In our opinion, multi-night stimulation may greatly boost the effectiveness of such interventions, but it will be important to think about dosage. Even a single night of TMR can lead to changes in brain structure and function that continue to unfold for at least 10 days [13], so the potential power of these techniques to sculpt the brain when applied across multiple nights should not be underestimated. While such interventions have the potential to impact positively on mental health, we should also acknowledge that improper and uninformed use over long periods of time could also impact on semantic and emotional processing in maladaptive ways. For instance, TMR might accidentally strengthen negative memories rather than weakening them, and could also trigger nightmares and negative episodes in those with PTSD. Furthermore, selectively boosting the consolidation of some memories and not others across multiple nights could lead to unnatural and skewed schemas. The warped predictions made by these incorrect internal models could potentially lead to confusion, and might even tip people who are at risk of schizophrenia over into clinical manifestations [14]. We therefore urge caution with respect to dosage and long-term application of TMR, as well as a broader understanding of these possible adverse effects.

Sleep engineering provides a powerful tool for the manipulation of both emotion and memory. We suggest that the use of EEG classifiers to quantify memory reactivation and examine its impact upon these aspects of cognition should ultimately give us the control and understanding we need to safely roll out these exciting new methods to human patients.

## Abbreviations

EEG: electroencephalogram
NREM: non-rapid eye movement
PTSD: post-traumatic stress disorder
REM: rapid eye movement
TMR: targeted memory reactivation
